# Highly Alkaline-Resistant *Enterococcus faecalis* Induces Compromised M1 Polarization and Phagocytosis of Macrophage via Z-DNA Binding Protein 1

**DOI:** 10.3390/microorganisms13122647

**Published:** 2025-11-21

**Authors:** Yifang Xiao, Runze Liu, Yanling Yang, Xinlu Li, Yi Min, Wei Fan

**Affiliations:** The State Key Laboratory of Oral & Maxillofacial Reconstruction and Regeneration, Key Laboratory of Oral Biomedicine Ministry of Education, Hubei Key Laboratory of Stomatology, School & Hospital of Stomatology, Wuhan University, 237 Luoyu Road, Wuhan 430079, China

**Keywords:** *Enterococcus faecalis*, alkaline resistance, M1 polarization, phagocytosis, Z-DNA binding protein 1

## Abstract

Persistent apical periodontitis (PAP) of human teeth is related to *Enterococcus faecalis* (*E. faecalis*) with higher alkaline resistance. This study aimed to investigate how highly alkaline-resistant (HAR) *E. faecalis* modulates macrophage M1 polarization and phagocytosis via Z-DNA-binding protein 1 (ZBP1). HAR *E. faecalis* was generated through serial alkaline passaging. RAW264.7 macrophages were infected with standard or HAR *E. faecalis*. M1 polarization markers and *Cd274* (Programmed death-ligand 1 (PD-L1)) were profiled by RT-qPCR. ZBP1 was detected by RT-qPCR and immunofluorescence staining, and was silenced using small interfering RNA (siRNA). iNOS, ZBP1 and PD-L1 proteins were analyzed by Western blotting. The phagocytosis of CFDA-SE-labeled bacteria was then quantified by confocal microscopy and flow cytometry. The results showed that HAR *E. faecalis* induced significantly lower expression of ZBP1, M1 polarization markers and *Cd274* in macrophages than the standard strain. After ZBP1 knock-down, expression of these markers decreased. Macrophages phagocytosed much fewer HAR *E. faecalis* than the standard strain. After ZBP1 knock-down, the differences between the two strains disappeared. In conclusion, HAR *E. faecalis* induced compromised M1 polarization and phagocytosis of macrophage via ZBP1. These findings may provide new insights into the pathogenesis and treatment of PAP.

## 1. Introduction

Persistent apical periodontitis (PAP) of human teeth refers specifically to a chronic pathological condition characterized by persistent or recurrent inflammation following root canal treatment. Although modern root canal treatment techniques exhibit a high success rate, PAP still occurs in approximately 30–40% of cases [1]. The primary etiological factor of PAP is the residual microbial biofilm within the root canal system and around the root apex, particularly *Enterococcus faecalis* (*E. faecalis*), which is detected in 24–77% of PAP cases and is frequently associated with intracanal calcium hydroxide (CH) medication [2]. Notably, *E. faecalis* isolated from the root canals of PAP patients shows high alkaline resistance [3,4]. This resistance enables their survival in the alkaline environment of CH-treated root canals, which may contribute to the pathogenesis of PAP [5]. However, why the alkaline-resistant *E. faecalis* is related to PAP and the differences in inflammation-inducing or suppressing potential between highly alkaline-resistant (HAR) and normal *E. faecalis* remains unclear.

Macrophages serve as central regulators in the immune response during PAP [6]. Recent studies have demonstrated that Z-DNA binding protein 1 (ZBP1), as a critical nucleic acid sensor [7], primarily activates downstream signaling pathways (particularly RIPK3-MLKL-mediated necroptosis [8], NLRP3 inflammasome [9,10] and NF-κB/IRF [11,12,13]) by sensing Z-RNA/Z-DNA derived from pathogens or endogenous damage signals [14,15], thereby playing a pivotal regulatory role in macrophage function. Macrophage polarization state directly influences disease progression: pro-inflammatory M1 macrophages exert antimicrobial effects through cytokines such as TNF-α and IL-6 [16], yet exacerbate bone tissue destruction [17,18]. The phagocytic function of macrophages serves as a critical defense mechanism for the host to eliminate bacterial infections [19,20]. However, it is unclear whether ZBP1 is involved in the inflammatory responses during HAR *E. faecalis* infection through regulating macrophage M1 polarization and phagocytosis.

Programmed death-ligand 1 (PD-L1), as a pivotal immune checkpoint molecule [21], plays a critical role in tumor immune escape [22] and chronic infections [23] by binding to the PD-L1 receptor to suppress T-cell activity [24] and to remodel macrophage function [25,26]. In the context of oral infections, PD-L1 levels are significantly elevated in the periodontal tissues of periodontitis patients [27] and are closely associated with the virulence factors of *Porphyromonas gingivalis* [28,29,30], suggesting that PD-L1 may serve as a target for pathogen-mediated immune escape. However, in PAP, the association of PD-L1 with persistent *E. faecalis* infection remains unknown. A study showed that ZBP1 also contributed to Unfolded Protein Response (UPR) activation during infection [12]. The UPR process is initiated by the endoplasmic reticulum trans-membrane protein inositol-requiring enzyme 1α (IRE1α/ERN1) [31]. Promoting PD-L1 expression through the IRE1α-XBP1 axis is beneficial for eliminating anti-tumor immunity [32]. It is worth investigating whether HAR *E. faecalis* affects PD-L1-dependent immunosuppression via modulating ZBP1.

This study aimed to compare the differential inflammatory effects between HAR and normal *E. faecalis* and investigate whether HAR *E. faecalis* influences the M1 polarization and phagocytic ability of macrophage via ZBP1.

## 2. Materials and Methods

### 2.1. Cell Culture

The murine RAW264.7 macrophage cell line ATCC SC-6003 (ATCC, Manassas, VA, USA) was maintained in Dulbecco’s Modified Eagle Medium (DMEM, GIBCO, Waltham, MA, USA) containing 10% fetal bovine serum (GIBCO, Waltham, MA, USA). For experimental procedures, cells were plated at a density of 1 × 10^6^ cells per well in six-well plates and allowed to adhere overnight prior to experimentation.

### 2.2. E. faecalis Culture

Prior to experimental applications, standard *E. faecalis* ATCC 29212 (ATCC, Manassas, VA, USA) was maintained in Brain Heart Infusion broth (BHI, Beijing Land Bridge Technology, Beijing, China) at 37 °C, 5% CO_2_, 1% O_2_.

### 2.3. Screening of HAR E. faecalis

**Figure 1:**
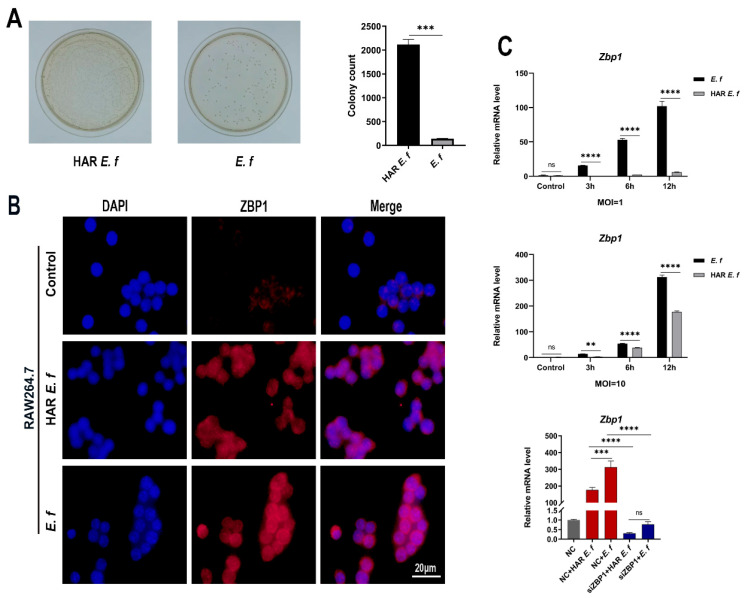
Activation of ZBP1 in macrophages by different *E. faecalis*. (**A**) The difference in CFUs of HAR and standard *E. faecalis* after being cultured in alkaline BHI for 24 h (Welch’s *t*-test). (**B**) Immunofluorescence staining of ZBP1 in HAR and standard *E. faecalis*-treated (MOI = 10, 12 h) cells (Scale bar, 20 μm). (**C**) mRNA levels of *Zbp1* in RAW264.7 cells infected with HAR and standard *E. faecalis* (MOI = 1, 10) for 3 h, 6 h and 12 h (Two-way ANOVA); and mRNA levels of *Zbp1* in RAW264.7 cells infected with HAR and standard *E. faecalis* (MOI = 10) for 12 h after *Zbp1* silencing (One-way ANOVA). NC, negative control. Data are expressed as the mean ±SD (** *p* < 0.01; *** *p* < 0.001; **** *p* < 0.0001; ns = no significant).

Exponential-phase standard *E. faecalis* suspensions (100 μL, OD_600_ = 1.0) were transferred to an alkaline (pH = 10) BHI medium containing 3 mg/mL CH [4]. Sequential subculturing (3 generations, 37 °C/24–48 h per cycle) under sustained alkaline stress yielded alkali-adapted variants that were purified by single-colony streaking on agar plates and designated as HAR *E. faecalis*. For phenotypic validation, equal quantities of HAR and standard *E. faecalis* were cultured separately in the same alkaline BHI medium mentioned above. After 24 h of incubation, 50 μL of liquid was plated on BHI agar plates and incubated at 37 °C for 24 h. High alkaline resistance was confirmed when HAR *E. faecalis* demonstrated a ≥1 log10 increase in colony-forming units (CFUs) relative to the standard strain (Figure 1A).

### 2.4. Cell Infection

Prior to microbial stimulation experiments, RAW264.7 cells were seeded in 6-well culture plates at a density of 1 × 10^6^ cells/well and cultured for 12 h, with one well reserved for cell counting. Concurrently, cryopreserved HAR and standard *E. faecalis* were revived and adjusted to the OD_600_ of 1.0 (approximately 1 × 10^9^ CFUs/mL). Bacterial suspensions were added into cells at the calculated volumes to achieve the target multiplicities of infection (MOI) of 1 and 10. Infection groups were cultured under 37 °C, 5% CO_2_ at three time points (3, 6, and 12 h). Cells without infection were used as control.

### 2.5. Cellular Immunofluorescence Staining

For immunofluorescence analysis, RAW264.7 macrophages were plated in 12-well chambers (5 × 10^5^ cells/well) and exposed to standard and HAR *E. faecalis* at MOI = 10 for 12 h. Post-infection processing included: (i) Fixation: 4% paraformaldehyde (PFA) in Phosphate-buffered saline (PBS) (20 min, room temperature); (ii) Permeabilization: 0.2% Triton X-100 (Beyotime, Shanghai, China; 10 min); (iii) Blocking: 5% BSA in PBS (30 min, 37 °C). Primary antibody incubation was conducted overnight at 4 °C with anti-ZBP1 (1:500, AG-20B-0010, AdipoGen, Liestal, Switzerland). Samples were subsequently incubated with fluorescently labeled secondary antibodies for 1 h, nuclei were counterstained with DAPI (Beyotime, Shanghai, China) and mounted in anti-fade mounting medium. Images were acquired on a Zeiss LSM880 Fast microscope (Zeiss, Oberkochen, Germany) via ZEN Blue 3.9 software.

### 2.6. Small Interfering RNA (siRNA)-Mediated Gene Silencing

ZBP1-targeting siRNA (siZBP1; GenePharma, Suzhou, China) was reverse-transfected into RAW264.7 cells using siRNA-mate transfection reagent (GenePharma, Suzhou, China) according to the manufacturer’s protocol. Parallel control groups received validated scrambled siRNA (NC-siRNA; GenePharma, Suzhou, China) at an equivalent concentration, and the knockdown result was confirmed by RT-qPCR and Western blot. The siRNA sequences are listed in Table 1.

### 2.7. Reverse Transcription Quantitative Real-Time PCR (RT-qPCR)

Cellular RNA isolation was conducted with TRIzol reagent (Life Technologies, Thermo Fisher Scientific, Waltham, MA, USA), followed by first-strand cDNA synthesis using ABScript III Reverse Transcriptase Master Mix (ABclonal, Wuhan, China). Quantitative amplification was performed in triplicate on a LightCycler 480 II platform (Roche Diagnostics, Basel, Switzerland) with SYBR Green master mix (Servicebio, Wuhan, China). Relative transcript quantification employed the comparative threshold cycle (2^−ΔΔCT^) method, with glyceraldehyde-3-phosphate dehydrogenase (GAPDH) serving as the endogenous normalization control. Primer sequences used in this experiment are listed in Table 2.

### 2.8. Western Blot Analysis

Cellular lysates were prepared using RIPA lysis buffer supplemented with protease inhibitor cocktail (both from Beyotime, Shanghai, China). Protein samples underwent denaturation (95 °C, 5 min) prior to electrophoretic separation on 10% SDS-PAGE systems (Servicebio, Wuhan, China). Wet transfer (200 mA, 60 min) immobilized proteins onto PVDF membranes (0.22 µm, Merck Millipore, Billerica, MA, USA), followed by 1 h blocking in 5% non-fat milk in Tris-Buffered Saline with Tween 20 (TBST). Membranes were probed overnight at 4 °C with the following validated antibodies: anti-iNOS (1:1000, T55993, Abmart, Shanghai, China), anti-ZBP1 (1:1000, AG-20B-0010, AdipoGen, Liestal, Switzerland), anti-PD-L1 (1:1000, M033179, Abmart, Shanghai, China) and anti-β-actin (1:1000, PMK058, BIOPRIMACY, Wuhan, China). After TBST washes (3 × 10 min), membranes were incubated with HRP-conjugated secondary antibody (1:5000, SA00001-1 or SA00001-2, Proteintech, Chicago, IL, USA) for 1 h at room temperature. Chemiluminescent detection was performed using ECL Prime (EpiZyme, Shanghai, China) with signal capture on an Odyssey System (LI-COR Biosciences, Lincoln, NE, USA). Densitometric analysis utilized ImageJ 1.53t (NIH, Bethesda, MD, USA).

### 2.9. Confocal Microscopy Imaging Analysis

Log-phase cultures of both standard and HAR *E. faecalis* were fluorescently labeled with CFDA-SE (Elabscience, Wuhan, China). The labeled bacteria were used to infect both control and *Zbp1*-knockdown murine macrophages in the log phase (grown on coverslips) at an MOI of 20 [33]. Infection was conducted at 37 °C for 40 min in the dark. Phagocytosis was terminated by incubating samples on ice for 10 min. A 0.04% trypan blue solution was applied for 1 min to quench fluorescence from extracellular bacteria. Samples were rinsed three times with PBS. Cells were fixed with 4% paraformaldehyde at room temperature for 20 min, followed by three PBS washes. Cells were incubated with TRITC-phalloidin (OriLeaf, Shanghai, China) for 30 min at room temperature in the dark, followed by PBS washes. Samples were mounted using anti-fade mounting medium containing DAPI (Beyotime, Shanghai, China). Processed samples were imaged using a confocal laser scanning microscope (CLSM, Zeiss LSM880, Carl Zeiss, Oberkochen, Germany).

### 2.10. Phagocytosis Assay

Both control and *Zbp1*-knockdown RAW264.7 cells were infected with CFDA-SE (Elabscience, Wuhan, China)-labeled standard or HAR *E. faecalis* (MOI = 20 [33]) at 37 °C for 40 min in the dark. Extracellular bacteria were quenched with 0.04% trypan blue. After three PBS washes, the cells were pelleted (200 × g, 5 min), resuspended in PBS, and analyzed on a CytoFLEX flow cytometer (Beckman Coulter Life Sciences, Indianapolis, IN, USA) using the FITC-A channel.

### 2.11. Statistical Analysis

All experiments were conducted in triplicate and data were presented as mean ± standard deviation (SD). Data analysis was performed using GraphPad Prism 8.0 software. Comparisons were made using Welch’s *t*-test, one-way analysis of variance (ANOVA), or two-way ANOVA. Statistical significance was set at *p* < 0.05 (* *p* < 0.05; ** *p* < 0.01; *** *p* < 0.001; **** *p* < 0.0001).

## 3. Results

### 3.1. HAR E. faecalis Induced Lower ZBP1 Expression in Macrophages than Standard Strain

**Figure 2:**
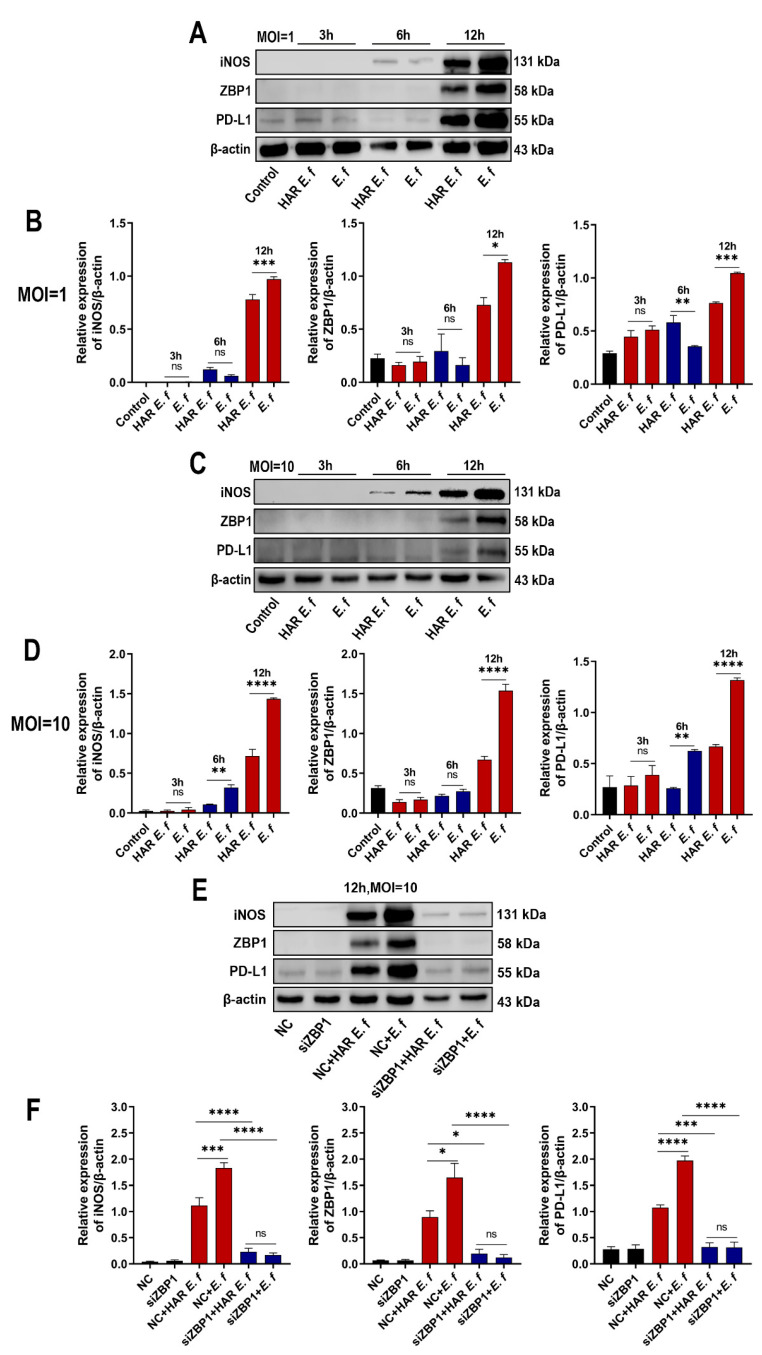
Macrophages stimulated with HAR *E. faecalis* showed compromised protein expression of iNOS and PD-L1 via ZBP1, relative to those stimulated with the standard strain. (**A**–**D**) Protein levels of iNOS, ZBP1 and PD-L1 in RAW264.7 cells infected with HAR and standard *E. faecalis* (MOI = 1, 10) for 3 h, 6 h and 12 h. The quantitative data represent the relative ratio of the target protein to β-actin (Two-way ANOVA). (**E**,**F**) Protein levels of iNOS, ZBP1 and PD-L1 in RAW264.7 cells infected with HAR and standard *E. faecalis* (MOI = 10) for 12 h after ZBP1 knockdown (NC, negative control). The quantitative data represent the relative ratio of the target protein to β-actin (One-way ANOVA). Data are expressed as the mean ±SD (* *p* < 0.05; ** *p* < 0.01; *** *p* < 0.001; **** *p* < 0.0001; ns = no significant).

Immunofluorescence results demonstrated that ZBP1 protein expression in RAW264.7 cells was upregulated upon both standard and HAR *E. faecalis* infection (Figure 1B). RT-qPCR and Western blot analyses confirmed that both standard and HAR *E. faecalis* infection upregulated ZBP1 expression when compared with the control group, while HAR *E. faecalis* induced significantly lower ZBP1 expression in macrophages than the standard *E. faecalis* (Figure 1C and Figure 2A, Figure 2B, Figure 2C and Figure 2D). Additionally, ZBP1 expression was higher at the MOI of 10 relative to the MOI of 1 (Figure 1C). Upon ZBP1 knockdown using siRNA, the ZBP1 levels activated by both standard and HAR *E. faecalis* were markedly reduced without difference between the two strains (Figure 1C and Figure 2E,F).

### 3.2. HAR E. faecalis Showed Compromised M1 Polarization Expression and Cd274(PD-L1) in Macrophages Through ZBP1

**Figure 3:**
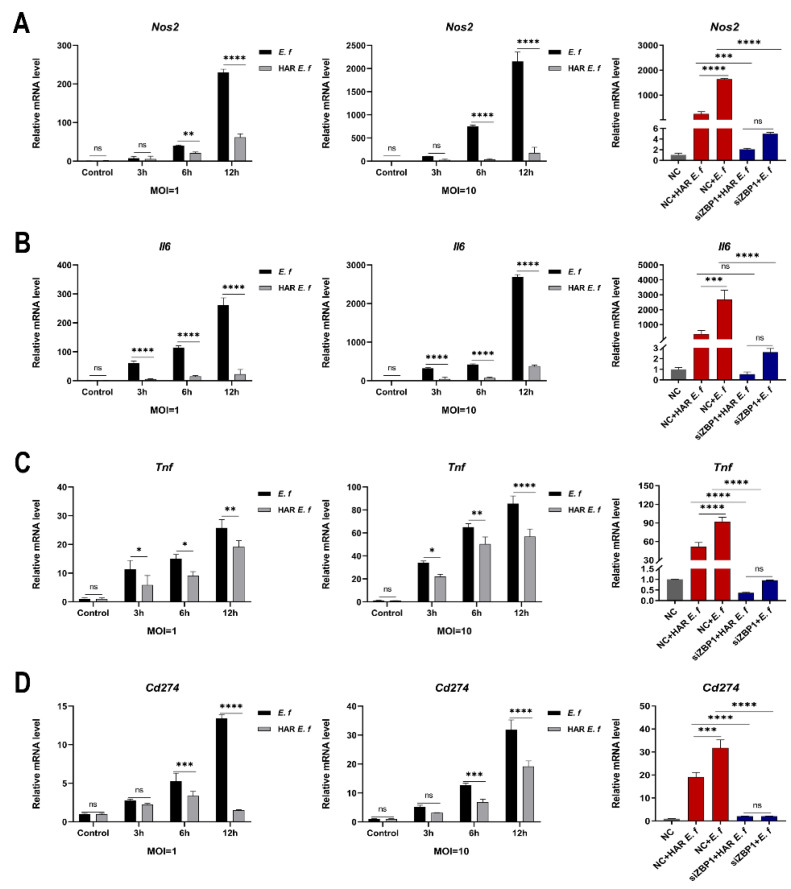
Macrophages stimulated with HAR *E. faecalis* showed compromised gene expression of M1 polarization markers and *Cd274* via *Zbp1*, relative to those stimulated with the standard strain. (**A**–**D**) mRNA levels of *Nos2*, *Il6*, *Tnf* and *Cd274* in RAW264.7 cells infected with HAR and standard *E. faecalis* (MOI = 1, 10) for 3 h, 6 h and 12 h (Two-way ANOVA); and mRNA levels of *Nos2*, *Il6*, *Tnf* and *Cd274* in RAW264.7 cells infected with HAR and standard *E. faecalis* (MOI = 10) for 12 h after *Zbp1* silencing (One-way ANOVA). NC, negative control. Data are expressed as the mean ±SD (* *p* < 0.05; ** *p* < 0.01; *** *p* < 0.001; **** *p* < 0.0001; ns = no significant).

RT-qPCR and Western blot analyses revealed that in RAW264.7 macrophages, both standard and HAR *E. faecalis* induced dose- and time-dependent upregulation of the M1 markers *Nos2* (iNOS), *Il6*, *Tnf* and the immune checkpoint gene *Cd274* (PD-L1), yet HAR *E. faecalis* showed significantly lower expressions of these genes than the standard strain ( Figure 2A–D and Figure 3). siRNA-mediated ZBP1 silencing eliminated the difference in expression of M1 markers and *Cd274* (PD-L1) induced by both HAR and standard *E. faecalis* and significantly reduced their overall expression levels (Figure 2E,F and Figure 3). 

### 3.3. HAR E. faecalis Compromised Macrophage Phagocytosis

**Figure 4:**
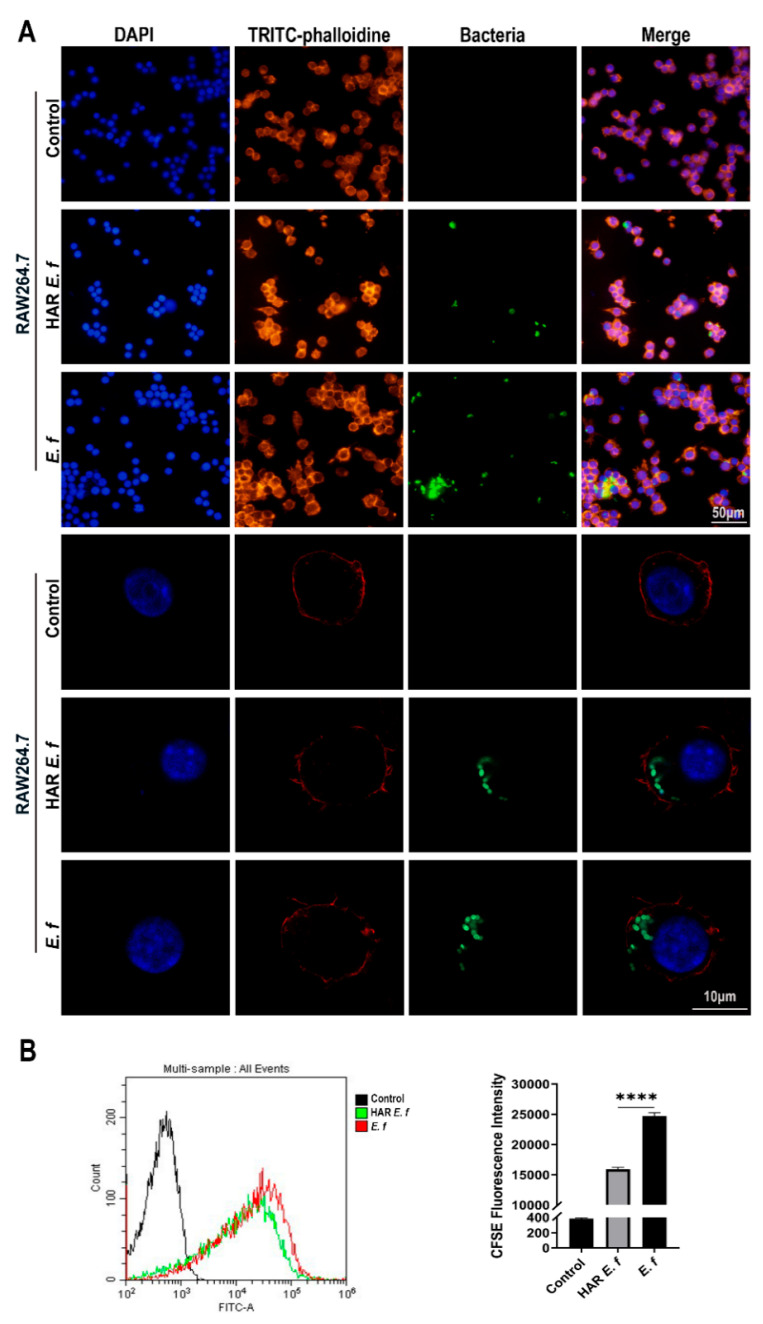
Phagocytosis of macrophages on standard and HAR *E. faecalis*. (**A**) Confocal microscopy images of macrophages infected with CFDA-SE-labeled HAR and standard *E. faecalis* (MOI = 20). (**B**) Flow cytometry plots of macrophages infected with CFDA-SE-labeled HAR and standard *E. faecalis* (MOI = 20; one-way ANOVA). Data are expressed as the mean ±SD (**** *p* < 0.0001).

**Figure 5:**
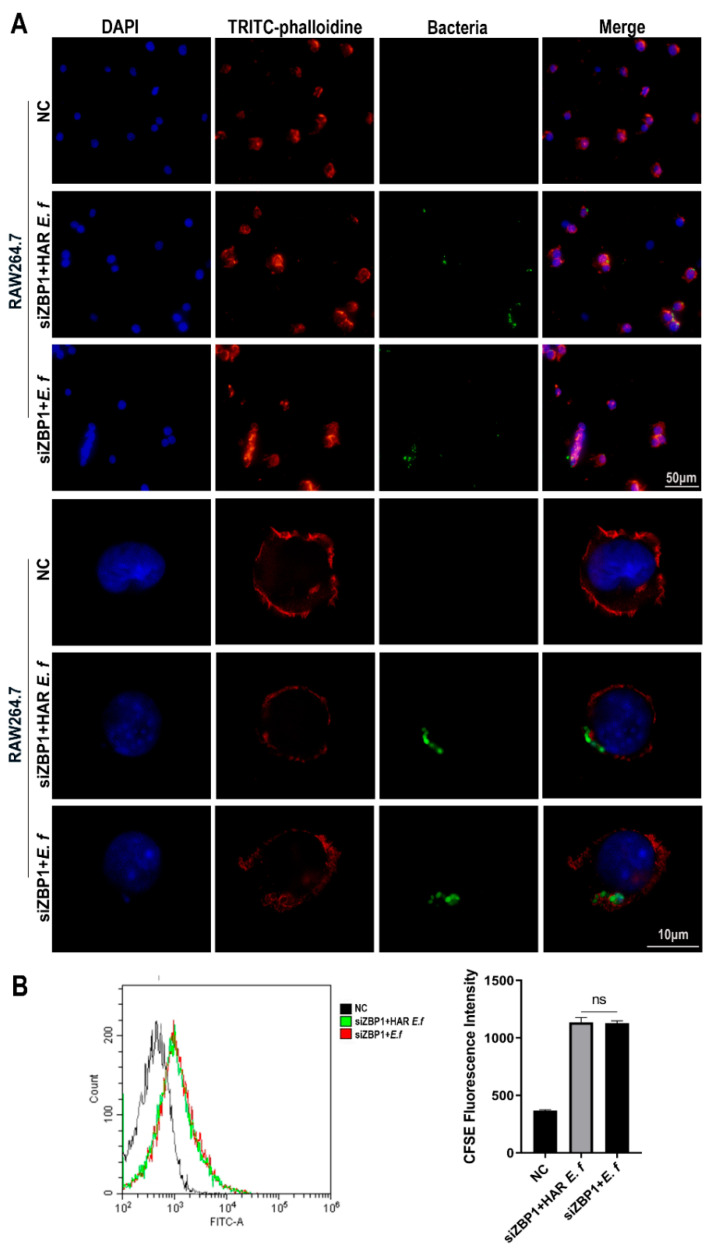
Phagocytosis of macrophages on standard and HAR *E. faecalis* after ZBP1 knockdown. (**A**) Confocal microscopy images of macrophages infected with CFDA-SE-labeled HAR and standard *E. faecalis* after ZBP1 knockdown (MOI = 20). (**B**) Flow cytometry plots of macrophages infected with CFDA-SE-labeled HAR and standard *E. faecalis* after ZBP1 knockdown (MOI = 20; one-way ANOVA). Data are expressed as the mean ±SD (ns = no significant).

Confocal microscopy and flow cytometric analysis revealed that both strains induced a typical M1-polarized morphology in macrophages, characterized by cell enlargement, extensive membrane ruffling, and pseudopodia formation after 40 min of infection at an MOI of 20 (Figure 4). The number of HAR *E. faecalis* internalized by RAW264.7 macrophages was significantly lower than that of the standard *E. faecalis*. Furthermore, following ZBP1 knockdown, phagocytosis of macrophage cells on both *E. faecalis* strains was attenuated without difference between the strains, and intracellular bacteria were significantly reduced (Figure 5).

## 4. Discussion

In PAP, *E. faecalis* has been identified as a related pathogen [34]. *E. faecalis*, especially those that survive root canal disinfection and medication procedures, often show alkaline resistance to CH [35], but the correlation between these alkaline-resistant *E. faecalis* and immune status in the apical tissues of PAP remains unclear. Macrophages serve as the primary cells responsible for recognizing and eliminating bacteria in periapical tissues [36], and their pro-inflammatory M1 polarization could regulate the progression of inflammation and promote pathogen removal [37]. The phagocytic ability of macrophages is also crucial for eliminating bacteria and can be influenced by bacteria [38]. Their innate immune sensor ZBP1 can influence pathogen clearance and tissue damage [39]. However, ZBP1’s role in PAP related to HAR *E. faecalis* is also unclear. Therefore, this study aimed to investigate the potential mechanism by which HAR *E. faecalis* affects macrophage M1 polarization and phagocytosis through regulating ZBP1 in macrophages.

Previous studies identified *F. nucleatum*-infected RAW264.7 cells polarized to the M1 phenotype, and this was accompanied by inflammatory cytokine production [40]. ZBP1 inhibition reduced inflammatory cytokine secretion and the occurrence of PANoptosis [41]. This study indicated that both HAR and standard *E. faecalis* can up-regulate ZBP1 in macrophages, exhibiting time-dependent and MOI-dependent patterns. However, the HAR strain showed a weaker ability to activate ZBP1 expression compared to the standard strain. Potential explanations for this phenomenon include unique genetic characteristics of the HAR strain which may encode specific proteins that interfere with ZBP1 transcription or signal transduction [9]. At the mechanistic level, ZBP1 can activate NF-κB and MAPK signaling pathways [42], promoting the massive release of inflammatory cytokines such as TNF-α and IL-6. These cytokines, in turn, drive macrophage polarization toward the M1 phenotype via autocrine or paracrine signaling, establishing a pro-inflammatory state [16]. In this study, the expression trends of M1 polarization markers were consistent with those of ZBP1, indicating that ZBP1 plays an important role in regulating macrophage polarization.

Recent studies have revealed that PD-L1 expression is not only regulated by the host microenvironment but can also be directly hijacked by pathogens [43]. *E. faecalis*, a pathogen related to post-endodontic treatment recurrence [44], may promote chronic infection through PD-L1-mediated suppression of both antimicrobial immunity and tissue repair [45], though the underlying molecular pathways remain unresolved. In this study, compared to the standard strain, HAR *E. faecalis* exhibited a unique “low immune activation-low immunosuppression” phenotype. The low level of immune activation compromises antigen presentation and co-stimulatory signals, thereby inhibiting the activation of antigen-specific T and B cells and ultimately preventing the host from developing robust, long-lasting immunity [46], which can lead to chronic or recurrent periapical infections. This study confirms that ZBP1 is a key molecule in inducing PD-L1 expression by HAR and standard strains, as its knockout leads to a significant reduction in PD-L1 expression. It is noteworthy that the HAR strain exhibits significantly weaker activation of the ZBP1-PD-L1 axis compared to the standard strain, yet they retain some PD-L1 induction capability even after ZBP1 knockout. This phenomenon may suggest that HAR strain could evade host surveillance by activating alternative pathways such as TLR2/MyD88 [47] or cGAS-STING [48] as a compensatory mechanism to the PD-L1.

Previous reports have demonstrated that bacterial surface modifications (e.g., capsular polysaccharides [49,50], protein conformation [51], or charge alterations [52]) can inhibit phagocytosis. The anti-phagocytic characteristics of *E. faecalis* involve synergistic actions of multiple mechanisms [53,54]. However, the specific molecular mechanisms linking its alkaline resistance to phagocytosis inhibition remain to be fully elucidated. In this study, comparative analysis of macrophage phagocytic capacity revealed a significantly lower efficiency in engulfing HAR *E. faecalis* compared to standard *E. faecalis*. During the early stage of infection, ZBP1 activates the NF-κB and MAPK signaling pathways [42], thereby enhancing phagocytic capacity [55,56]. In this study, ZBP1 knock down resulted in reduced phagocytic capacity of macrophages, further confirming its critical role in early immunity. Furthermore, HAR *E. faecalis* induced compromised expression of PD-L1 and phagocytosis via ZBP1, thereby facilitating immune evasion. Similar mechanisms have been reported in methicillin-resistant *Staphylococcus aureus* [57,58], suggesting that different pathogens may employ analogous strategies to achieve immune evasion.

Although this study indicated the mechanistic role of ZBP1 in macrophage-mediated inflammatory responses to HAR *E. faecalis* infection, several limitations remain to be addressed. First, the mechanisms by which HAR *E. faecalis* regulates ZBP1 expression, and how this differs from the standard *E. faecalis* strain, remain incompletely understood. It is unclear whether this regulation involves Toll-like receptor signaling or alterations in bacterial surface proteins. Second, systematic comparative analyses are needed to determine whether there are intrinsic differences in surface protein profiles between HAR and standard *E. faecalis* strains. Third, the molecular basis underlying the interaction between ZBP1 and macrophage phagocytic capacity needs further investigations. In particular, it remains to be elucidated whether ZBP1-mediated regulation is directly controlled by specific transcription factors or mediated through intermediate signaling molecules. Future studies should prioritize deciphering the molecular “inflammatory switch” mechanism governed by ZBP1 and explore the development of therapeutic strategies targeting specific inflammatory pathways to control refractory infections involving HAR *E. faecalis*.

## 5. Conclusions

HAR *E. faecalis* induced compromised M1 polarization and significantly impaired the phagocytic capacity of macrophages compared with the standard strain, thereby likely enhancing its capacity for immune evasion. These findings may provide new insights into the pathogenesis and treatment of PAP of human teeth.

## Figures and Tables

**Table 1:** siRNA sequences for gene knockdown.

Gene	Forward (5′-3′)	Reverse (5′-3′)
siZBP1	GGACAUAGAAAGCUCUCAATT	UUGAGAGCUUUCUAUGUCCTT
Negative control	UUCUCCGAACGUGUCACGUTT	ACGUAGCACGUUCGGAGAATT

**Table 2:** Primer sequences used for the RT-qPCR.

Gene	Forward (5′-3′)	Reverse (5′-3′)
*Zbp1*	AAGAGTCCCCTGCGATTATTTG	TCTGGATGGCGTTTGAATTGG
*Nos2*	TCAATGGGACTGCATATCTGCC	GCCAAAATACTACCAGCTCACT
*Il6*	TAGTCCTTCCTACCCCAATTTCC	TTGGTCCTTAGCCACTCCTTC
*Tnf*	TATGGCCCAGACCCTCACA	GGAGTAGACAAGGTACAACCCATC
*Cd274*	TCAGCTACGGTGGTGCGGACT	AGCTTCTGGATAACCCTCGGCCT
*Gapdh*	TGTGTCCGTCGTGGATCTGA	TTGCTGTTGAAGTCGCAGGAG

## Data Availability

The original contributions presented in this study are included in the article. Further inquiries can be directed to the corresponding authors.

## Abbreviations

ANOVA	Analysis of Variance
ATCC	American Type Culture Collection
BSA	Bovine Serum Albumin
CFDA-SE	Carboxyfluorescein Diacetate Succinimidyl Ester
CFU	Colony-Forming Unit
CH	Calcium Hydroxide
CLSM	Confocal Laser Scanning Microscopy
DAPI	4′,6-Diamidino-2-Phenylindole
DMEM	Dulbecco’s Modified Eagle Medium
*E. faecalis*	*Enterococcus faecalis*
FBS	Fetal Bovine Serum
GAPDH	Glyceraldehyde-3-Phosphate Dehydrogenase
HAR	Highly Alkaline-Resistant
HRP	Horseradish Peroxidase
iNOS	Inducible Nitric Oxide Synthase
IRE1α/ERN1	Inositol-Requiring Enzyme 1α
MOI	Multiplicity of Infection
PAP	Persistent Apical Periodontitis
PBS	Phosphate-Buffered Saline
PD-L1	Programmed Death-Ligand 1
PFA	Paraformaldehyde
PVDF	Polyvinylidene Fluoride
RT-qPCR	Reverse Transcription Quantitative real-time Polymerase Chain Reaction
SD	Standard Deviation
SDS-PAGE	Sodium Dodecyl Sulfate–Polyacrylamide Gel Electrophoresis
siRNA	Small Interfering RNA
TBST	Tris-Buffered Saline with Tween 20
UPR	Unfolded Protein Response
ZBP1	Z-DNA Binding Protein 1
